# Hemisphere specific EEG related to alternate nostril yoga breathing

**DOI:** 10.1186/s13104-017-2625-6

**Published:** 2017-07-24

**Authors:** Shirley Telles, Ram Kumar Gupta, Arti Yadav, Shivangi Pathak, Acharya Balkrishna

**Affiliations:** Patanjali Research Foundation, Patanjali Yogpeeth, Maharishi Dayanand Gram, Bahadrabad, Haridwar, Uttarakhand 249402 India

**Keywords:** EEG, Alternate nostril yoga breathing, Cerebral hemisphere symmetry, Breath awareness, Quiet sitting, EEG relative power, EEG bands

## Abstract

**Background:**

Previously, forced unilateral nostril breathing was associated with ipsilateral, or contralateral cerebral hemisphere changes, or no change. Hence it was inconclusive. The present study was conducted on 13 normal healthy participants to determine the effects of alternate nostril yoga breathing on (a) cerebral hemisphere asymmetry, and (b) changes in the standard EEG bands.

**Methods:**

Participants were randomly allocated to three sessions (a) alternate nostril yoga breathing (ANYB), (b) breath awareness and (c) quiet sitting, on separate days. EEG was recorded from bilaterally symmetrical sites (FP_1_, FP_2_, C_3_, C_4_, O_1_ and O_2_). All sites were referenced to the ipsilateral ear lobe.

**Results:**

There was no change in cerebral hemisphere symmetry. The relative power in the theta band was decreased during alternate nostril yoga breathing (ANYB) and the beta amplitude was lower after ANYB. During quiet sitting the relative power in the beta band increased, while the amplitude of the alpha band reduced.

**Conclusion:**

The results suggest that ANYB was associated with greater calmness, whereas quiet sitting without specific directions was associated with arousal. The results imply a possible use of ANYB for stress and anxiety reduction.

## Background

The nasal cycle is an ultradian rhythm characterized by alternating congestion and decongestion of opposite nostrils [1]. The nasal mucosal membrane has innervation from the autonomic nervous system so that sympathetic dominance on one side results in nasal mucosal vasoconstriction hence increasing nostril patency on that side. On the contralateral side there would be parasympathetic dominance and nasal mucosal vasodilation resulting in partial or complete occlusion of the nostril on that side. The nasal cycle varies widely in periodicity. When a continuous recording of nostril dominance was made, time series analysis detected periods of the nasal cycle at 280–275, 165–210, 145–160, 105–140, 70–100 and 40–65 min bins [2, 3].

Werntz et al. [4] showed that the nasal cycle was also related to the function of the central nervous system. The finding that forced uninostril breathing has selective effects on the EEG of the cerebral hemispheres was first shown in 1983 and later on with greater rigor in 1987 [5]. This is believed to be due to a neural connection arising from the superior nasal meatus [6]. Activation of the upper nasal cavity could be produced by air insufflation without inflation of the lung [6]. Also local anesthesia of the local mucosal membrane prevented the cortical changes which follow upper nasal cavity activation.

In a comparison between forced uninostril breathing and bilateral breathing, the peak power of beta2 in the frontal EEG was lower during uninostril compared to bilateral breathing [7].

The effects of forced alternate nostril breathing on the EEG were studied in 18 trained persons who practiced forced alternate nostril breathing for 10 min [8]. The study aimed at differentiating between forced alternate nostril breathing which began with inhalation through the left nostril compared to forced alternate nostril with right nostril inhalation to begin with [8]. No difference was reported. However the average power in the beta and alpha bands increased during both types of forced alternate nostril breathing. Also during the latter half of the ten minutes of forced alternate nostril breathing there was a decrease in hemisphere asymmetry in the beta 1 band, which the authors described as ‘a balancing effect on the functional activity of the left and right hemisphere.’

Yoga voluntarily regulated breathing (*pranayama*) allows a practitioner to breathe through one nostril at a time, effortlessly and selectively [9]. Alternate nostril breathing is also a common yoga breathing practice [10]. In Indian medicine importance is given to uninostril and alternate nostril breathing [11]. The effects of uninostril breathing are described in detail, with left nostril breathing described as ‘cooling and ‘calming’, while right nostril breathing is described as ‘heat generating’ and energizing’, and alternate nostril breathing has been described as ‘harmonizing’ [11].

A previous study showed that 18 min of alternate nostril breathing lowered the systolic and diastolic blood pressure in persons with essential hypertension controlled by medication [12].

The present study was planned as a preliminary study to assess the effects of alternate nostril yoga breathing on the EEG.

The hypothesis of the present study was that alternate nostril yoga breathing would reduce hemisphere asymmetry in EEG as was observed for forced alternate nostril breathing.

## Methods

### Participants

Thirteen healthy males with ages between 18 and 45 years residing in a yoga center in north India participated in the study. They were recruited by flyers on the notice boards of the yoga center. To be included in the trial, participants had to meet the following criteria: (a) the participants had to have experience of yoga breathing (*pranayama*) of at least 45 min a day, practiced for at least 15 days per month, over a minimum period of 6 months, and (b) the participants all had to be right hand dominant based on a standard handedness questionnaire [13]. The exclusion criteria were (1) persons on any medication, and (2) the presence of any illness, particularly psychiatric or neurological disorders. None of the participants were excluded based on these criteria. The baseline characteristics of the participants are given in Table 1.

**Table 1:** Baseline characteristics of the participants (n = 13)

Age in years (group mean ± SD)	24.2 ± 4.7 years
Average years of education (group mean ± SD)	13.8 ± 1.6 years
Experience of yoga breathing including ANYB (group mean ± SD)	38.8 ± 32.6 months
Experience of ANYB exclusively	29.2 ± 22.8 months

*ANYB* alternate nostril yoga breathing

The experimental procedure was approved by the ethical committee of Patanjali Research Foundation and signed informed consent was obtained from each participant before beginning the study.

### Design of the study

The participants were assessed before, during and after the intervention. Each participant was assessed in three sessions, conducted on 3 separate days, keeping the time of the day constant for a particular participant. The three sessions were (a) alternate nostril yoga breathing (ANYB), (b) breath awareness (BAW), and (c) quiet sitting (QS). Participants were randomly assigned to the three sessions using a standard randomizer [14], hence the order of the three sessions was different for different participants.

**Fig. 1:**
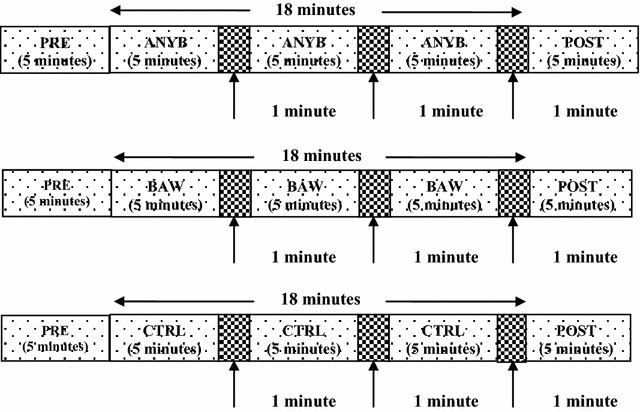
A schematic representation of the study design. The *stippled area* represents pre, during, and post periods. The *gray area* represents gaps between practice epochs

The total duration of each session was 28 min, i.e., 5 min before the practice, 18 min during the practice, and 5 min after the practice. During the practice the participants practiced ANYB, BAW or quiet sitting for 15 min with 1 min of rest after every 5 min of practice, so that the duration was 18 min. Hence the 15 min were divided into three epochs of 5 min each. Throughout the session participants were seated on a chair with their spine straight and eyes closed. Recordings were taken continuously in the pre, during 1, during 2, during 3 and post periods of 5 min each as shown in Fig. 1.

### Recording procedure

EEG was recorded using Ag/AgCl disc electrodes. The scalp was prepared using Nuprep skin preparation gel (Weaver and Co., USA). Electrodes with Ten20 conductive EEG Paste (Weaver and Co., USA) were placed at FP_1_, C_3_, and O_1_ referenced to the left ear lobe (A_1_), and at FP_2_, C_4_, and O_2_ referenced to the right ear lobe (A_2_); based on the standard 10–20 system for electrode placement [15]. Participants were seated in a dimly lit, sound and electrical-noise attenuated cabin adjacent to the recording room. Participants were able to receive instructions or communicate with the examiner using a two way intercom. Throughout a session participants were observed on a closed circuit television, which they were informed about prior to the session.

EEG was recorded using Neurotravel LIGHT (ATES Medica Device, Italy). The sampling frequency was 250 samples per second. The low cut filter was set at .2 Hz and the high cut filter at 30.0 Hz. This had the obvious limitation of not including gamma frequencies, which could not be recorded with this equipment.

### Interventions

#### Alternate nostril yoga breathing

The participants sat comfortably with their spine erect and shoulders relaxed with eyes closed. ANYB involves breathing through left and right nostrils alternately without retention of the breath. In this practice the thumb and the ring figure of the right hand were used to manipulate or occlude the nostrils [16]. Participants were asked to sit erect in either the half-lotus posture (*ardha*-*padmasana*) or complete lotus posture *(padmasana*). They were asked to keep their eyes closed, gently, without effort. After this they were asked to keep their non-dominant hand (the left hand in all participants) on their left knee. They flexed the right arm at the elbow and raised their right hand to the level of their nose. The index and middle fingers of the right hand were flexed to rest their fingertips on their palms, using their thumb and ring figure of the right hand to manipulate or occlude the nostrils [16]. Occlusion of the nostrils was gentle. Participants were asked to begin the breathing practice by exhaling through the left nostril with the right nostril occluded with the right thumb; then inhaling slowly through the left nostril; followed by exhaling through the right nostril with the left nostril occluded with the right ring finger; then inhaling through the right nostril and exhaling through the left nostril. With this exhalation one cycle was complete. The approximate duration of 1 cycle was 6 s; with the ratio of inhale:exhale as 1:1.5 [9]. Participants were asked to continue breathing like this for 5 min. This was timed by the yoga instructor. They were then given 1 min gap during which participants were asked to remain with their eyes closed and to rest their right fingers on their right knee. This (5 min followed by 1 min) was repeated thrice in the session.

#### Breath awareness

During breath awareness, the participants maintained awareness of the breath without manipulation of the nostrils. Participants were asked to sit erect in either the half-lotus (*ardha padmasana*) or complete lotus (*padmasana*) posture and keep their eyes closed. During this time both arms were extended and the hands were placed on the respective knees. The instructor asked the participants to direct their attention to the movement of air into and out of their nostrils and also direct their awareness to the movement of air through the nasal passages. The period of breath was 5 min, timed by the instructor, followed by instructions to allow attention to wander for 1 min. This (5 min followed by 1 min) was repeated thrice in the session.

#### Quiet sitting

Participants were asked to sit with their spine erect and shoulders relaxed with eyes closed. Participants were asked to keep their eyes closed and to sit in either the half-lotus (*ardha padmasana*) or complete lotus posture (*padmasana*). They were asked to stretch their arms out to rest the fingers of each hand on the respective knees. Participants were told to allow their thoughts to wander without restrictions. After 5 min they were told there was a 1 min gap, though the instructions during the 1 min gap did not differ from the 5 min preceding it. This (5 and 1 min gap) was repeated thrice in the session.

### Data extraction

EEG records were visually inspected for artifacts due to eye or body movements. The recordings were all free from artifact and no part of the records had to be excluded for analysis. The artifact-free data were spectrally analyzed using fast Fourier transform analysis (FFT). This analysis provided the relative power for each band as a percentage of the total power. This was provided for the delta (.5–3.5 Hz), theta (4–7.5 Hz), alpha (8–12) and beta (13–30 Hz) bands. Also, the actual values of the average amplitude within a band for a specific period (e.g., before alternate nostril yoga breathing) were obtained. These values were used for analysis.

### Data analysis

Statistical analysis was carried out using SPSS (Version 18.0). Repeated measures analyses of variance (RM-ANOVA) were performed with two within subjects factors, i.e., Sessions (ANYB, BAW and QS), and States (pre, during, and post). An ANOVA was followed by post hoc tests for multiple comparisons with Bonferroni adjustment.

The Bonferroni adjustment was carried out for each of the multiple post hoc comparisons. The comparisons which were considered were the ‘during’ and ‘post’ values compared with the ‘pre’ values of a specific session. This was separate for each EEG band. With the SPSS software Bonferroni adjustment multiplies the uncorrected p value by the number of comparisons; hence α remains unchanged [17].

## Results

### Repeated-measures analysis of variance

1. Energy of the EEG bands as a percentage of the whole
  The theta energy (%) at C_4_ − A_2_ and O_2_ − A_2_ showed a significant difference between States (p < .05; F = 2.730, df = 1, 48; p < .05; F = 1.868, df = 1, 48 respectively). The beta energy (%) at FP_2_ − A_2_ showed a significant difference between States (p < .05; F = 4.482, df = 1, 48).
2. Amplitudes of the EEG bands
  The beta amplitude at O_2_ − A_2_ showed a significant difference between States (p < .05; F = 8.400, df = 1, 48). The alpha amplitude at C_4_ − A_2_ showed a significant difference between States (p < .05; F = .676, df = 1, 48).

For all comparisons the Huynh–Feldt epsilon was equal to 1.000, hence sphericity was assumed.

### *Post*-*hoc* analyses with Bonferroni adjustment

The theta energy (%) was significantly reduced at C_4_ − A_2_, and O_2_ − A_2_ during the practice of ANYB compared to the values before the practice (p < .05), for both comparisons. In contrast there was a significant increase in the beta energy (%) at FP_2_ − A_2_ sites during QS compared to before QS (p < .05).

There was a significant reduction in the beta amplitude at O_2_ − A_2_ after the practice of ANYB compared to before ANYB (p < .05). During the QS session there was a significant reduction in the alpha amplitude at C_4_ − A_2_ compared to before QS (p < .05).

**Fig. 2:**
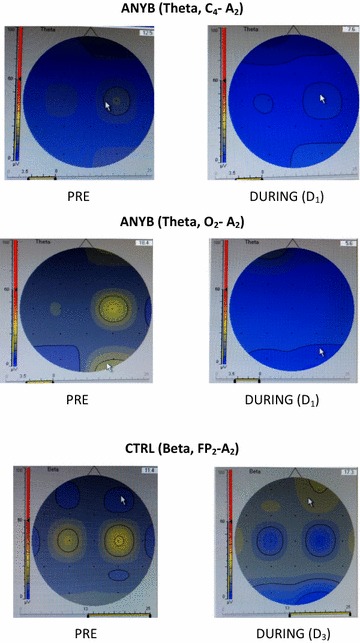
Energy of the theta and beta bands (μV^2^). Energy of the theta band (μV^2^) showing a significant reduction at C_4_ − A_2_ and O_2_ − A_2_ during alternate nostril yoga breathing compared to before. Energy of the beta band increased during quiet sitting compared to before at FP_2_ − A_2_

**Fig. 3:**
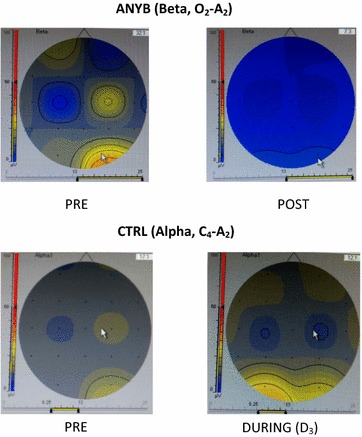
Amplitudes of the beta and alpha bands (µV). Amplitude of the beta band (μV) showing a significant reduction at O_2_ − A_2_ during alternate nostril yoga breathing compared to before. Amplitude of the alpha band decreased during quiet sitting compared to before at C_4_ − A_2_

There were no significant changes following breath awareness. The mean values ± SD for energy (%) and amplitude at FP_1_ − A_1_, FP_2_ − A_2_, C_3_ − A_1_, C_4_ − A_2_, O_1_ − A_1_, and O_2_ − A_2_ electrode sites pre, during and post ANYB, BAW and QS are provided in Tables 2, 3 and 4. Significant changes in EEG energy (%) and EEG amplitude are shown in Figs. 2 and 3, respectively.

**Table 2:** Energy (%) of the four EEG bands (μV^2^) pre, during and post, ANYB, BAW and QS sessions

Sl. no.	Bands	ANYB					BAW					QS				
		Pre M ± SD	During			Post M ± SD	Pre M ± SD	During			Post M ± SD	Pre M ± SD	During			POST M ± SD
			D1 M ± SD	D2 M ± SD	D3 M ± SD			D1 M ± SD	D2 M ± SD	D3 M ± SD			D1 M ± SD	D2 M ± SD	D3 M ± SD	
FP_1_ − A_1_	Delta	31.5 ± 9.6	29.4 ± 12.3	28.9 ± 13.9	28.5 ± 12.2	27.5 ± 11.2	25.2 ± 10.4	24.0 ± 10.4	21.5 ± 10.1	25.5 ± 10.2	26.0 ± 10.4	29.6 ± 10.5	28.0 ± 11.9	28.9 ± 13.9	33.5 ± 15.9	30.8 ± 12.8
	Theta	4.2 ± 2.7	2.8 ± 1.5	4.4 ± 4.0	3.5 ± 2.2	5.0 ± 4.1	3.6 ± 3.2	3.8 ± 2.8	4.0 ± 3.9	3.9 ± 2.6	3.7 ± 2.0	4.0 ± 3.5	4.2 ± 4.1	4.4 ± 4.0	4.3 ± 3.2	4.1 ± 3.3
	Alpha	2.6 ± 1.5	2.9 ± 2.6	2.1 ± 1.5	3.7 ± 4.0	3.0 ± 2.3	3.4 ± 5.5	3.3 ± 4.8	3.6 ± 6.7	4.4 ± 7.2	3.7 ± 5.1	2.4 ± 2.0	2.2 ± 1.7	2.1 ± 1.5	2.3 ± 1.5	.2 ± 1.3
	Beta	.8 ± .4	.8 ± .4	.8 ± .6	1.0 ± .5	1.2 ± .8	.8 ± .8	.8 ± .6	.8 ± .6	1.0 ± .7	.9 ± .7	.7 ± .5	.7 ± .6	.8 ± .6	.7 ± .5	.8 ± .6
FP_2_ − A_2_	Delta	28.4 ± 11.4	26.0 ± 11.1	27.3 ± 14.2	26.7 ± 12.9	25.6 ± 10.3	25.7 ± 9.0	24.2 ± 10.7	21.8 ± 11.3	25.0 ± 11.0	25.7 ± 11.5	27.8 ± 9.4	26.3 ± 12.2	27.3 ± 14.2	29.3 ± 12.1	30.1 ± 12.6
	Theta	3.8 ± 2.4	2.8 ± 1.2	4.8 ± 4.4	4.0 ± 2.5	4.8 ± 3.6	4.1 ± 3.1	4.8 ± 3.6	4.6 ± 4.8	4.7 ± 3.4	4.3 ± 2.6	4.5 ± 3.6	4.8 ± 5.1	4.8 ± 4.4	5.0 ± 3.4	4.8 ± 3.5
	Alpha	2.8 ± 1.8	3.2 ± 2.7	2.4 ± 1.8	4.6 ± 5.0	3.2 ± 2.5	3.7 ± 4.4	3.8 ± 4.0	3.5 ± 4.9	4.7 ± 6.1	4.1 ± 4.6	3.0 ± 2.6	2.6 ± 2.1	2.4 ± 1.8	2.8 ± 1.7	2.8 ± 1.7
	Beta	.6 ± .2	.7 ± .2	.7 ± .5	.2 ± 1.0	.9 ± .5	.7 ± .4	.8 ± .5	.8 ± .6	.9 ± .5	.9 ± .6	.7 ± .4	.7 ± .5	.7 ± .5	.8 ± .5*	.8 ± .5
C_3_ − A_1_	Delta	22.7 ± 6.1	21.5 ± 6.2	24.3 ± 5.5	20.9 ± 7.5	23.7 ± 6.5	21.3 ± 5.7	20.8 ± 7.3	21.7 ± 7.4	22.5 ± 8.1	24.2 ± 8.9	21.9 ± 5.0	23.4 ± 4.0	24.3 ± 5.5	24.7 ± 5.8	23.9 ± 5.3
	Theta	9.6 ± 4.2	8.0 ± 3.0	10.4 ± 4.1	8.6 ± 3.3	10.4 ± 3.0	10.3 ± 6.6	9.4 ± 4.3	9.7 ± 4.4	9.8 ± 4.3	10.5 ± 4.9	9.1 ± 4.2	10.0 ± 4.2	10.4 ± 3.0	10.0 ± 4.1	8.6 ± 3.3
	Alpha	14.3 ± 12.2	13.6 ± 11.6	13.5 ± 11.3	16.5 ± 16.4	16.3 ± 15.1	16.1 ± 15.0	15.6 ± 16.2	16.2 ± 16.8	16.3 ± 16.8	17.2 ± 17.4	15.1 ± 14.1	14.8 ± 12.5	13.5 ± 11.3	13.3 ± 10.4	13.4 ± 11.6
	Beta	2.7 ± 1.5	2.6 ± 1.4	2.9 ± 1.5	3.1 ± 1.6	2.9 ± 1.4	2.6 ± 1.4	2.5 ± 1.5	2.6 ± 1.4	2.7 ± 1.5	2.7 ± 1.4	2.6 ± 1.8	2.8 ± 1.4	2.8 ± 1.5	2.8 ± 1.4	2.9 ± 1.5
C_4_ − A_2_	Delta	24.6 ± 8.1	22.2 ± 8.1	26.8 ± 6.1	21.1 ± 8.9	24.7 ± 7.4	23.7 ± 4.9	23.0 ± 7.0	24.9 ± 7.2	26.1 ± 7.8	26.1 ± 7.8	24.7 ± 5.7	24.8 ± 5.5	26.8 ± 6.1	26.3 ± 5.3	26.6 ± 6.1
	Theta	10.4 ± 4.2	8.2 ± 3.4*	11.5 ± 4.2	8.2 ± 3.4	10.6 ± 2.5	10.9 ± 4.1	11.0 ± 4.6	11.3 ± 3.9	11.8 ± 4.4	11.6 ± 4.6	4.2 ± 10.7	4.2 ± 11.0	3.0 ± 11.5	11.0 ± 3.8	11.1 ± 4.0
	Alpha	15.4 ± 13.0	14.9 ± 13.5	13.2 ± 10.2	15.9 ± 14.3	14.7 ± 11.8	17.6 ± 15.2	16.1 ± 14.5	17.0 ± 15.2	16.8 ± 15.5	17.0 ± 16.2	14.8 ± 12.5	13.3 ± 10.6	13.2 ± 10.2	12.6 ± 10.0	12.5 ± 8.9
	Beta	3.3 ± 1.8	3.7 ± 1.7	2.8 ± .9	4.2 ± 2.8	3.6 ± 1.9	3.2 ± 1.7	2.9 ± 1.3	2.9 ± 1.2	2.8 ± 1.3	2.9 ± 1.2	2.8 ± 1.1	3.0 ± 1.4	2.8 ± .9	2	2.8 ± 1.0
O_1_ − A_1_	Delta	17.8 ± 5.3	17.2 ± 6.5	19.1 ± 6.6	17.5 ± 6.5	20.0 ± 8.2	16.8 ± 6.4	19.6 ± 10.2	18.3 ± 8.9	18.7 ± 9.0	20.1 ± 9.0	17.7 ± 6.3	17.5 ± 5.4	19.1 ± 6.6	19.2 ± 7.2	20.1 ± 6.8
	Theta	6.7 ± 2.9	5.8 ± 3.4	7.2 ± 3.9	6.0 ± 2.9	7.7 ± 2.9	6.5 ± 3.2	7.2 ± 4.0	7.1 ± 4.1	7.5 ± 4.2	7.8 ± 4.2	7.0 ± 3.8	6.7 ± 3.4	7.2 ± 3.9	6.9 ± 3.7	7.2 ± 3.7
	Alpha	21.1 ± 18.2	18.3 ± 17.4	15.4 ± 14.4	21.2 ± 21.0	19.1 ± 18.1	19.1 ± 18.2	18.1 ± 20.2	18.0 ± 20.1	18.5 ± 21.4	18.7 ± 20.8	19.2 ± 17.1	17.3 ± 15.6	15.4 ± 14.4	16.5 ± 15.8	17.2 ± 15.0
	Beta	2.9 ± 1.6	2.9 ± 2.1	2.4 ± 1.0	2.9 ± 2.0	3.2 ± 1.8	2.6 ± 1.3	2.7 ± 1.6	2.7 ± 1.5	2.6 ± 1.4	2.6 ± 1.3	2.5 ± 1.2	2.5 ± 1.2	2.4 ± 1.0	2.4 ± 1.0	2.4 ± .9
O_2_ − A_2_	Delta	19.7 ± 8.8	18.1 ± 7.9	20.9 ± 7.3	18.1 ± 7.4	21.5 ± 8.4	17.7 ± 5.1	19.5 ± 7.6	19.5 ± 5.6	20.1 ± 8.4	19.8 ± 7.5	20.3 ± 7.1	20.1 ± 5.6	20.9 ± 7.3	20.9 ± 7.6	20.7 ± 7.3
	Theta	7.2 ± 2.7	5.8 ± 2.8*	8.6 ± 4.7	6.1 ± 2.2	9.3 ± 4.2	7.0 ± 3.3	7.8 ± 3.6	7.9 ± 2.6	8.1 ± 3.6	7.7 ± 3.2	8.0 ± 3.7	9.0 ± 5.0	8.6 ± 4.6	7.9 ± 4.4	8.2 ± 4.3
	Alpha	20.8 ± 17.9	18.7 ± 17.2	16.3 ± 15.8	20.8 ± 17.4	19.0 ± 14.7	19.8 ± 21.1	17.5 ± 19.8	19.7 ± 20.1	18.3 ± 20.4	17.8 ± 20.0	18.6 ± 17.1	19.0 ± 17.8	16.3 ± 15.8	16.9 ± 16.6	17.7 ± 16.9
	Beta	2.8 ± 1.3	2.8 ± 1.2	2.6 ± 1.4	2.9 ± 1.1	2.8 ± 1.1	2.5 ± 1.2	2.7 ± 1.5	2.8 ± 1.5	2.6 ± 1.4	2.5 ± 1.4	2.6 ± 1.2	2.7 ± 1.3	2.6 ± 1.4	2.4 ± 1.1	2.4 ± 1.1

Comparisons were of post and during values compared with the pre values of the respective session, i.e., ANYB, BAW and QS. p < .05, RM ANOVA, followed by post hoc tests with Bonferroni adjustment

*ANYB* alternate nostril yoga breathing, *BAW* breath awareness, *QS* quiet sitting

**Table 3:** Amplitudes of the four EEG bands (in μV) pre, during and post, ANYB, BAW and QS sessions

Sl. no.	Band	ANYB					BAW					QS				
		Pre M ± SD	During			Post M ± SD	Pre M ± SD	During			Post M ± SD	Pre M ± SD	During			Post M ± SD
			D1 M ± SD	D2 M ± SD	D3 M ± SD			D1 M ± SD	D2 M ± SD	D3 M ± SD			D1 M ± SD	D2 M ± SD	D3 M ± SD	
FP_1_ − A_1_	Delta	35.3 ± 16.8	35.0 ± 12.1	35.6 ± 19.5	31.2 ± 12.5	30.0 ± 15.1	34.3 ± 17.9	30.3 ± 15.4	32.2 ± 21.6	28.9 ± 14.8	28.7 ± 13.4	39.0 ± 20.0	35.4 ± 17.1	35.6 ± 19.5	37.4 ± 27.2	35.7 ± 19.1
	Theta	12.1 ± 7.3	10.3 ± 3.2	12.3 ± 9.2	10.5 ± 3.5	11.1 ± 5.4	11.7 ± 8.7	11.8 ± 7.2	11.2 ± 6.1	9.9 ± 3.9	9.8 ± 4.6	12.8 ± 8.3	12.0 ± 8.0	12.3 ± 9.2	12.5 ± 8.8	11.7 ± 7.0
	Alpha	9.1 ± 4.0	9.3 ± 4.3	8.3 ± 4.8	9.9 ± 5.3	8.5 ± 5.2	8.8 ± 4.4	8.4 ± 4.7	9.0 ± 6.0	8.6 ± 5.1	8.0 ± 4.5	9.1 ± 4.4	8.4 ± 4.5	8.3 ± 4.8	8.3 ± 4.7	8.3 ± 4.4
	Beta	5.0 ± 1.9	5.3 ± 1.9	5.0 ± 3.0	5.7 ± 2.8	5.5 ± 3.3	4.9 ± 2.3	4.8 ± 1.9	5.3 ± 3.1	4.9 ± 2.3	4.8 ± 2.2	5.1 ± 2.7	5.1 ± 3.1	5.0 ± 3.0	4.9 ± 2.9	5.0 ± 2.7
FP_2_ − A_2_	Delta	31.0 ± 13.7	30.8 ± 11.6	32.6 ± 19.2	27.0 ± 10.9	26.8 ± 13.3	28.6 ± 12.3	24.8 ± 9.8	29.4 ± 21.1	24.9 ± 12.7	24.5 ± 10.3	34.4 ± 15.8	32.9 ± 17.6	32.6 ± 19.2	33.1 ± 24.6	31.7 ± 16.6
	Theta	11.3 ± 5.9	10.0 ± 2.9	12.2 ± 8.5	10.0 ± 3.2	10.5 ± 4.8	10.6 ± 6.1	10.2 ± 4.1	11.2 ± 6.0	9.8 ± 3.7	9.4 ± 3.6	12.4 ± 7.2	11.9 ± 7.4	12.2 ± 8.5	12.1 ± 7.8	11.5 ± 6.4
	Alpha	9.0 ± 3.9	9.4 ± 4.3	8.6 ± 5.2	10.0 ± 5.4	8.5 ± 5.1	8.8 ± 4.4	8.5 ± 4.7	9.2 ± 6.3	8.9 ± 5.6	8.1 ± 4.4	9.6 ± 4.9	8.9 ± 4.9	8.6 ± 5.2	8.7 ± 5.1	8.7 ± 5.1
	Beta	4.4 ± 1.1	5.0 ± 1.4	4.8 ± 2.2	5.8 ± 2.4	4.8 ± 2.9	4.6 ± 2.0	4.3 ± 1.2	4.8 ± 2.5	4.4 ± 1.7	4.3 ± 1.3	4.9 ± 2.0	5.0 ± 2.5	4.8 ± 2.2	4.8 ± 2.3	4.8 ± 2.1
C_3_ − A_1_	Delta	14.3 ± 2.0	15.4 ± 3.1	14.8 ± 2.8	14.6 ± 2.1	14.0 ± 1.8	14.1 ± 2.2	14.1 ± 2.4	14.7 ± 4.4	14.1 ± 2.9	13.9 ± 3.0	15.7 ± 2.9	14.8 ± 2.4	14.8 ± 2.8	15.0 ± 3.4	14.8 ± 2.8
	Theta	9.4 ± 1.6	9.2 ± 1.9	9.7 ± 2.5	9.6 ± 2.1	9.4 ± 1.9	9.2 ± 1.9	9.5 ± 2.1	9.8 ± 3.3	9.4 ± 2.3	9.0 ± 1.8	9.9 ± 2.5	9.6 ± 2.7	9.7 ± 2.5	9.6 ± 2.6	9.7 ± 2.8
	Alpha	10.1 ± 5.6	10.8 ± 6.2	10.6 ± 6.8	11.9 ± 7.4	11.0 ± 7.1	10.5 ± 5.8	10.6 ± 6.5	11.2 ± 7.4	10.9 ± 6.9	10.1 ± 6.2	11.6 ± 6.9	10.9 ± 6.6	10.6 ± 6.8	10.6 ± 6.2	10.7 ± 6.7
	Beta	4.8 ± 1.3	5.2 ± 1.5	5.1 ± 2.1	5.7 ± 1.7	4.9 ± 1.6	4.9 ± 1.5	4.9 ± 1.6	5.1 ± 2.1	4.8 ± 1.6	4.6 ± 1.5	5.2 ± 2.0	5.1 ± 1.8	5.1 ± 2.1	5.1 ± 1.9	5.2 ± 1.9
C_4_ − A_2_	Delta	14.7 ± 2.0	15.3 ± 2.8	14.9 ± 3.3	15.1 ± 2.9	14.1 ± 2.5	14.7 ± 2.2	14.9 ± 3.3	14.8 ± 3.5	15.0 ± 3.1	14.4 ± 2.4	15.1 ± 3.1	14.9 ± 3.4	14.9 ± .3.3	15.1 ± 4.8	15.0 ± 3.6
	Theta	9.5 ± 1.8	9.3 ± 2.1	9.7 ± 2.5	9.7 ± 2.0	9.4 ± 1.8	9.9 ± 2.3	10.1 ± 2.6	10.2 ± 3.1	10.2 ± 2.8	9.6 ± 2.1	9.8 ± 2.3	9.7 ± 2.5	9.7 ± 2.5	9.5 ± 2.4	9.5 ± 2.4
	Alpha	10.8 ± 5.2	11.9 ± 5.8	10.3 ± 6.0	13.0 ± 6.7	10.7 ± 6.1	11.5 ± 6.4	11.5 ± 7.2	12.0 ± 7.9	11.6 ± 7.6	10.9 ± 6.8	11.2 ± 6.0	10.5 ± 5.7	10.3 ± 6.0	10.2 ± 5.6*	10.2 ± 5.6
	Beta	5.6 ± 2.6	6.8 ± 3.4	5.2 ± 1.6	7.6 ± 5.1	5.5 ± 2.2	5.2 ± 1.4	5.1 ± 1.8	5.2 ± 1.9	5.0 ± 1.9	4.9 ± 1.6	5.2 ± 1.8	5.2 ± 1.9	5.2 ± 1.6	4.9 ± 1.5	5.0 ± 2.0
O_1_ − A_1_	Delta	12.9 ± 3.7	16.0 ± 7.8	14.0 ± 4.8	17.1 ± 7.6	12.8 ± 3.4	13.0 ± 2.9	13.2 ± 3.8	13.1 ± 3.8	13.1 ± 4.4	12.1 ± 2.5	13.3 ± 14.7	14.7 ± 6.4	14.0 ± 4.8	14.5 ± 6.2	14.4 ± 5.7
	Theta	8.0 ± 2.7	8.8 ± 4.3	8.4 ± 2.8	9.3 ± 4.0	7.9 ± 2.8	8.1 ± 2.6	8.3 ± 2.6	8.1 ± 2.5	8.2 ± 2.8	7.8 ± 3.1	8.5 ± 2.5	8.4 ± 2.7	8.4 ± 2.8	8.2 ± 2.6	8.3 ± 3.0
	Alpha	13.5 ± 10.1	14.7 ± 11.2	12.3 ± 9.6	15.6 ± 12.3	12.6 ± 11.0	12.9 ± 8.6	12.5 ± 9.9	13.0 ± 9.8	12.5 ± 10.2	11.7 ± 9.7	13.5 ± 9.2	13.6 ± 9.8	12.3 ± 9.6	12.2 ± 8.9	12.5 ± 9.1
	Beta	5.2 ± 2.2	6.3 ± 3.9	5.0 ± 2.0	6.2 ± 3.0	5.1 ± 2.7	5.2 ± 1.8	5.1 ± 1.9	5.1 ± 2.2	4.9 ± 2.0	4.6 ± 2.0	5.2 ± 2.1	5.1 ± 2.0	5.0 ± 2.0	4.9 ± 1.9	4.9 ± 2.0
O_2_ − A_2_	Delta	13.2 ± 3.7	15.0 ± 6.0	13.9 ± 4.2	14.1 ± 4.1	12.3 ± 2.7	12.3 ± 1.7	12.1 ± 2.1	12.0 ± 2.8	12.5 ± 3.1	11.8 ± 1.8	14.1 ± 3.7	13.7 ± 4.0	13.9 ± 4.2	14.0 ± 4.9	14.2 ± 5.0
	Theta	8.3 ± 2.5	8.6 ± 2.9	8.7 ± 3.3	8.5 ± 2.6	8.2 ± 2.4	7.7 ± 1.7	7.8 ± 2.1	7.9 ± 2.4	7.9 ± 2.1	7.6 ± 2.0	8.7 ± 3.1	8.6 ± 3.4	8.7 ± 3.3	8.3 ± 3.0	8.5 ± 3.4
	Alpha	13.7 ± 9.2	15.0 ± 10.4	11.9 ± 10.5	15.3 ± 10.7	12.2 ± 9.1	11.6 ± 7.1	10.9 ± 7.1	11.4 ± 7.4	11.1 ± 7.3	10.6 ± 6.9	13.3 ± 9.9	12.5 ± 9.9	11.9 ± 10.5	11.7 ± 9.1	12.2 ± 9.8
	Beta	5.2 ± 2.4	6.2 ± 3.3	4.8 ± 2.0	5.9 ± 2.5	4.7 ± 2.1*	4.6 ± 1.4	4.5 ± 1.5	4.5 ± 1.7	4.5 ± 1.6	4.3 ± 1.4	5.2 ± 2.2	4.9 ± 1.9	4.8 ± 2.0	4.7 ± 1.9	4.7 ± 2.1

Comparisons were of post and during values compared with the pre values of the respective session, i.e., ANYB, BAW and QS. p < .05, RM ANOVA, followed by post hoc tests with Bonferroni adjustment

*ANYB* alternate nostril yoga breathing, *BAW* breath awareness, *QS* quiet sitting

**Table 4:** Left right coherence as a measure of hemisphere asymmetry, recorded at prefrontal, vertex and occipital sites in ANYB, BAW and QS sessions

Sl. no.	FP_1_ − A_1_ and FP_2_ − A2 (max)		C_3_ − A_1_ and C_4_ − A_2_ (max)		O_1_ − A1 and O_2_ − A_2_ (max)		FP_1_ − A1 and FP_2_ − A_2_ (2-peck)		C_3_ − A1 and C_4_ − A_2_ (2-peck)		O_1_ − A_1_ and O_2_ − A_2_ (2-peck)	
	Mean	SD	Mean	SD	Mean	SD	Mean	SD	Mean	SD	Mean	SD
ANYB												
Pre	.92	.04	.89	.03	.79	.07	.87	.05	.86	.04	.72	.07
D1	.90	.05	.87	.05	.76	.10	.85	.08	.84	.05	.69	.10
D2	.90	.05	.88	.03	.78	.07	.85	.07	.85	.04	.71	.07
D3	.90	.05	.88	.04	.77	.09	.85	.08	.84	.05	.70	.09
Post	.91	.04	.90	.03	.79	.07	.91	.18	.87	.03	.73	.06
BAW												
Pre	.90	.06	.89	.03	.78	.06	.87	.07	.86	.04	.72	.07
D1	.89	.06	.88	.03	.78	.05	.90	.23	.85	.03	.73	.06
D2	.90	.05	.89	.03	.79	.06	.86	.06	.86	.03	.73	.05
D3	.89	.05	.89	.03	.78	.05	.85	.05	.85	.03	.72	.05
Post	.89	.06	.95	.22	.79	.05	.85	.06	.96	.41	.73	.05
QS												
Pre	.93	.05	.89	.03	.80	.06	.89	.06	.85	.03	.74	.05
D1	.91	.05	.89	.03	.80	.06	.87	.06	.85	.04	.75	.05
D2	.91	.05	.88	.04	.80	.05	.87	.06	.85	.04	.74	.06
D3	.91	.05	.88	.03	.79	.06	.87	.06	.85	.03	.74	.06
Post	.92	.06	.89	.03	.80	.06	.88	.07	.85	.05	.75	.06

*ANYB* alternate nostril yoga breathing, *BAW* breath awareness, *QS* quiet sitting

## Discussion

Contrary to the hypothesis of the study alternate nostril yoga breathing was not associated with any change in cerebral hemisphere EEG symmetry. The relative power in the theta band reduced during alternate nostril yoga breathing (ANYB), while the amplitude of beta waves was lower after ANYB. During the control period of quiet sitting (QS) the relative power in the beta band increased, while the amplitude of the alpha band reduced.

Hemispheric symmetry was determined (1) based on coherence as calculated by the software (Neurotravel, Italy), and (2) based on changes in the EEG amplitude recorded at symmetrical pre-frontal, vertex, and occipital sites over the left and the right hemispheres. As mentioned contrary to the hypothesis, alternate nostril yoga breathing did not alter hemispheric symmetry.

Changes in the relative power in the EEG bands occurred during ANYB and during quiet sitting. There was a decrease in the relative power in the theta band during ANYB at the vertex on the right side. Frontal theta activity has been related to working memory [18] and increased frontal and midline theta were related to a positive emotional state [19]. In general, variations in the power of theta and alpha bands of the EEG are related to complex cognitive functions and memory performance [20]. Hence the decrease in relative theta power may be associated with a better ability to perform certain cognitive tasks, though the connection is not strong. The theta activity increases in several conditions including drowsiness associated with a decreased ability to perform specific tasks [20].

The increase in relative power of the beta band of the EEG during quiet sitting over the right prefrontal region could suggest increased alertness, arousal and excitement, which are associated with increased beta wave activity [21]. Conversely, the amplitude of the beta wave band was lower after ANYB recorded over the right occipital region. Beta wave activity is not well understood, and its functional role remains only partially explained [22]. For instance research has shown that increased beta wave activity generated in the motor cortex is related to slow motor behavior [23]. A decrease of beta wave power (i.e., desynchronization) is believed to be an indicator of movement preparation, execution, and motor imagery [24, 25]. An arousal based theory [26] may help explain the changes in beta activity found in the present study. The arousal theory suggests that increased beta activity is associated with increased mental activity or arousal [26]. This suggests that after ANYB there is a decrease in arousal consistent with descriptions of yoga breathing as calming [8]. During the quiet sitting session, in contrast, the decrease in alpha amplitude over the right vertex could suggest greater arousal associated with random thinking in the absence of specific instructions [27]. This finding of increased activation during quiet sitting has been found in other studies [28]. It was suggested that the mental state during quiet sitting may be comparable to the state of mind wandering and self-referential processing [29].

Most of the changes described above (during and after ANYB, and during QS) occurred on the right side. These results may be considered comparable to those of an earlier study which assessed cerebral hemisphere specific task performance in 135 participants, aged between 10 and 17 years [30]. Participants were randomly assigned to (1) left nostril breathing, (2) right nostril breathing, (3) alternate nostril breathing, (4) breath awareness or (5) a control state. Hence there were 5 groups (n = 27 each) who practiced the intervention they were assigned to for 10 days. At the beginning and end of the 10 day period participants were assessed using verbal and spatial memory tasks, considered specific for left and right hemispheric functions, respectively. All four active intervention groups (left, right and alternate nostril yoga breathing as well as breath awareness) showed a significant increase by 84% in spatial memory scores at the end of 10 days. These results suggested that yoga breathing increases right hemisphere task performance. In the present study it is possible that during quiet sitting the participants who were trained in *pranayama* practiced yoga breathing inadvertently. It remains unclear why the breath awareness sessions showed no change unlike the study cited above. A possible reason is the small sample size which is a limitation of the study. Also, the present study assessed EEG, while the study cited above [30] assessed verbal and spatial memory task performance. It would have been ideal to record both measurements simultaneously. Hence simultaneous recording of the EEG and cognitive tasks could be a definite direction for future research.

The findings of the present study are limited by (a) the small sample size (n = 13; effect size = .11 (low), and (b) the inability to record and report the gamma band of the EEG with the equipment used.

Despite these limitations, this may be considered a pilot study which has results suggesting that ANYB may be calming and may possibly influence cognitive functions.

## Conclusions

Contrary to the hypothesis of the study there was no change in cerebral hemisphere asymmetry during alternate nostril yoga breathing. Alternate nostril yoga breathing resulted in a decrease in theta band energy at the vertex and occipital sites on the right side. There was a decrease in the amplitude of the beta band after alternate nostril yoga breathing at the right occipital site, while the amplitude of the alpha band reduced during sitting quietly without specific instructions at the right vertex site. Also during sitting quietly without specific instructions there was an increase in energy in the beta band at the right prefrontal site.

### Importance and relevance

Airflow through the nostril can impact the EEG. In this case alternate nostril yoga breathing had effects on the EEG suggesting that the practice can be calming and reduce arousal.

## Abbreviations

A_1_: reference (left ear lobe)
A_2_: reference (right ear lobe)
ANYB: alternate nostril yoga breathing
BAW: breath awareness
C_3_: vertex, left side
C_4_: vertex, right side
QS: quiet sitting
EEG: electroencephalography
FFT: fast Fourier transform
FP_1_: left pre-frontal
FP_2_: right pre-frontal
M: mean
O_1_: left occipital
O_2_: right occipital
RM-ANOVA: repeated measures analysis of variance
SD: standard deviation
